# Visceral Leishmaniasis and Land Use and Cover in the Carajás Integration Region, Eastern Amazon, Brazil

**DOI:** 10.3390/tropicalmed7100255

**Published:** 2022-09-22

**Authors:** Claudia do Socorro Carvalho Miranda, Bruna Costa de Souza, Tainara Carvalho Garcia Miranda Filgueiras, Alder Mourão de Sousa, Maira Cibelle da Silva Peixoto, Tainã Carvalho Garcia Miranda Filgueiras, Frederico José Carvalho Miranda, Sérgio Luiz Althoff, Raimundo Gladson Corrêa Carvalho, Nelson Veiga Gonçalves

**Affiliations:** 1 Laboratory of Epidemiology and Geoprocessing of Amazon, Para State University, Belém 66087-662, Brazil; 2 Superior School of Amazon, Belém 66053-180, Brazil; 3 Cyberspace Institute, Federal Rural University of Amazon, Belém 66077-830, Brazil; 4 Programa de Pós-graduação em Direito, Federal University of Para, Belém 66075-110, Brazil; 5 Animal Biology Laboratory, Natural Sciences Department, Blumenau Regional University, Blumenau 89012-078, Brazil

**Keywords:** visceral leishmaniasis, epidemiology, environment, spatial analysis, parasitology, vector-borne diseases, change in land-use

## Abstract

Human visceral leishmaniasis is a major public health problem in the Amazon. Thus, we analyzed the spatial distribution of this disease and its relationship with epidemiological, socioeconomic, and environmental variables in the Carajás Integration Region, Pará state, from 2011 to 2020. Epidemiological data for this ecological study were obtained from the State Public Health Secretariat, environmental data were obtained from the National Space Research Institute, and socioeconomic data were obtained from the Brazilian Geography and Statistics Institute. ArcGIS 10.5.1 software was used for classifying land use and cover and for the Kernel and Moran spatial analyses. It was observed in 685 confirmed cases that the epidemiological profile followed the national pattern of the disease occurrence, with a high prevalence in children who were not school-aged. The disease had a non-homogeneous distribution with clusters related to different human activities, such as urbanization, ranching, and mining. A spatial dependence between the disease prevalence and socioeconomic indicators was observed. The municipalities presented gradients of case densities associated with a direct relationship between areas with cases and deforestation. The disease is developing due to risk factors such as establishment and maintenance related to the non-sustainable development model implemented in the region, pointing to the need for its revision.

## 1. Introduction

Human visceral leishmaniasis (HVL) is an infectious disease caused by Trypanosomatid protozoans of the genus Leishmania, transmitted by female phlebotomid flies of the genus Lutzomyia. In urban environments, the main reservoir for this disease is the dog (*Canis familiaris* Lineu, 1758), while foxes (*Lycalopex vetulus* Lund, 1842 and *Cerdocyon thous* Linnaeus, 1766) and white-eared opossums (*Didelphis albiventris* Lund, 1840) are the main reservoirs in the wild [1]. Diagnosis is a complex process, based on clinical signs, epidemiological, hematological, and biochemical parameters [1].

According to the World Health Organization (2020) HVL has been notified in approximately 83 countries around the world. An estimated 30,000 new cases of the disease occur every year, and it is suspected that there is a major epidemiological silence, especially in East Africa [2]. It is endemic in 13 countries in the Americas, with 97% of the cases described being in Brazilian territory. The disease affects socially vulnerable individuals, and its occurrence has been associated with malnutrition, migratory processes, inadequate housing, difficulties with access to health services, and deforestation [1,2,3].

In Brazil, 2.529 new HVL cases were confirmed in 2019, with a prevalence rate of 1.2 cases per 100 thousand inhabitants and a lethality rate of 9%, the highest recorded over the last 10 years. The disease occurred in all regions of Brazil, with autochthonous cases in 24 states [3,4]. During the period of 2017 to 2019, the states of Maranhão, Pará, Minas Gerais, and Ceará recorded the largest numbers of cases, and in recent decades there have been changes in the HVL transmission pattern—it was initially notified in rural areas but has recently been reported in periurban and urban areas in Brazil [1,5,6].

To contain dissemination of the disease in Brazilian territory, the Ministry of Health has implemented the Visceral Leishmaniasis Surveillance and Control Program (VLSCP), which involves control measures based on early diagnoses and treatment of human cases, reduction of the vector population, and control of canine reservoir hosts and health education activities. However, this disease still poses a major public health problem because of several factors, including the multifactorial epidemiological relationships involved in its transmission chain and the different human actions developed in the national territory [1,2,3,4].

In terms of the Amazon region, the dynamics of deforestation and the intensification of land use and occupation in an unsustainable way have contributed towards leaving local populations more vulnerable to risk factors for sickness due to HVL [7,8]. There are difficulties in implementing measures for controlling the disease because of several factors, such as the great diversity and constant changes in epidemiological transmission factors, caused by the different species of vectors, reservoirs, and etiological agents, associated with human impacts on the environment [1,2,3,4]. In 2019, the large Eastern Amazon state of Pará confirmed 306 HVL cases in its territory, and according to the most recent data from the Brazilian Ministry of Health, the disease was notified in all regions of the state [9]. 

One of them, the Carajás Integration Region (CIR) located in the southeastern Pará mesoregion, has undergone major socioeconomic changes over the last few decades due to the arrival of major operations in mining (iron, copper, nickel, and manganese), agriculture and ranching (soy, corn, and cattle ranching), and infrastructure (ports, highways, river routes, and railroads) in its territory. That region, which accounts for 57% of the state’s industrial Gross Domestic Product (GDP) and medium Municipal Human Development Index (MHDI) in the order of 0.610, also had an intense migratory flow, environmental transformations, and an increase in the number of cases of HVL, highlighting the need for epidemiological monitoring of the region [9,10,11,12,13,14].

However, despite major potential for morbidity and mortality of the disease, there are still few studies that present epidemiological and environmental analysis for the contrasting realities in Pará regions such as the CIR. In that regard, geotechnology is being increasingly used for research in health issues and in elucidating the epidemiology of infectious and parasitic diseases through integrating a significant volume of cartographic information [15,16]. Geotechnology makes it possible to describe the geographic distribution of leishmaniasis and its risk factors and contribute towards a holistic and systemic view of its determinant and conditioning factors.

In light of the above, and seeking the construction of a contextualized scenario of Human Visceral Leishmaniasis, this study sought to analyze its spatial distribution and relation with epidemiological, socioeconomic, and environmental variables in the Carajás Integration Region, state of Pará, from 2011 to 2020.

## 2. Materials and Methods

**Figure 1:**
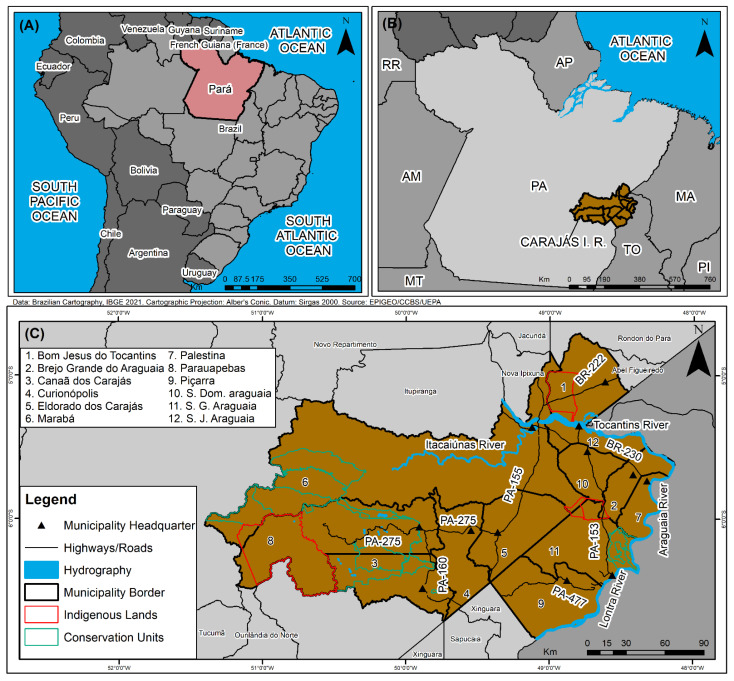
Spatial location of Pará ((**A**), red polygon), Carajás Integration Region ((**B**), brown polygon), and CIR municipalities map (**C**), Pará, Brazil.

A cross-sectional and ecological study was performed on confirmed HVL cases in the CIR for the period from 2011 to 2020. This region, considered in this work as the analysis spatial unity, is composed by 12 municipalities (Marabá, Parauapebas, Eldorado dos Carajás, Canaã dos Carajás, São Geraldo do Araguaia, São Domingos do Araguaia, Curionópolis, Bom Jesus do Tocantins, São João do Araguaia, Piçarra, Palestina do Pará and Brejo Grande do Araguaia) (Figure 1).

Epidemiological data (sex, age range, ethnicity, education, zone, type of entry, and evolution) were obtained from the Disease Notification Information System (SINAN) of the Ministry of Health. Cartographic data (municipal boundaries and seats, indigenous lands, and conservation units), demographic, and socioeconomic databases (populations, GDP and MHDI) were obtained from the Brazilian Geographic and Statistics Institute (IBGE) information systems. The environmental databases related to deforestation and land use and cover (pasture, agriculture, urban area, forest, mining, secondary vegetation, deforestation, and other classes) used in this study were obtained from the Brazilian Amazon Forest Monitoring Program by Satellite (PRODES) and from the Land Use and Cover Mapping Project in the Legal Amazon of the Brazilian National Institute for Space Research (INPE).

The data obtained were debugged to remove inconsistencies and incompleteness using Tabwin 36b software (Brazilian Ministry of Health, Brasilia—Federal District, Brazil). Next, confirmed cases of HVL were georeferenced in the field using a Global Positioning System (GPS) and later stored in a Geographic Database (BDGEO). During fieldwork it was possible to confirm deforestation in the study area, thus validating the data obtained. For the descriptive analysis, percentage calculations and the non-parametric chi-squared statistical test were used, with expected equal proportions with a significance of 0.05%. Considering the increase in the number of HVL cases and the increase in deforestation in the CIR municipalities over the study period, an analysis of linear trend between these two variables was performed. Both analyses were made using the Bioestat 5.0 program (Brazilian Ministry of Health, Brasilia—Federal District, Brazil).

The prevalence, MHDI, and GDP indicators of the CIR municipalities were shown in choropleth maps. The spatial distribution analysis of the disease to identify possible concentrations of cases and their evolution over time, at two 5-year intervals (2011 to 2015 and 2016 to 2020), used Kernel Density Estimation (KDE). For construction of the thematic map of land use and cover the forest, hydrography, secondary vegetation, deforestation, agriculture, mining, pasture, and “other classes” (non-observed area) classes were considered. To assess the spatial correlation between areas with deforestation and those cases of HVL, the Moran Global Bivariate index (I) was employed. To that end, the hypotheses of “inverse” (I < 0), “random” (I = 0), and “direct” (I > 0) spatial autocorrelation were accepted, with a significance at *p* < 0.05. A strong spatial correlation was considered if (I) was close to one of the variation limits (−1, 1). Arcgis 10.5.1 software (ESRI, Redlands, CA, USA) was used for all the analyses above.

Ethical requirements were followed in this study in line with the Helsinki Declaration, the Nuremberg Code, and the rules in Resolution no. 466/12 of the National Health Council. This study received favorable opinion 3.292.673 from the Research Ethics Committee of Pará State University.

## 3. Results

During the study period, 685 HVL cases were confirmed in the CIR, with Marabá (197), Parauapebas (164), Eldorado dos Carajás (145), and Canaã dos Carajás (89) reporting greater numbers of disease cases, followed by São Geraldo do Araguaia (34), Curionópolis (31), São Domingos do Araguaia (8), Piçarra (6), São João do Araguaia (4), Bom Jesus do Tocantins (4), and Palestina do Pará (3). The municipality of Brejo Grande do Araguaia did not present cases.

The distribution of percentages of HVL cases in all months of the years of the study period occurred with values close to the average (61.1), except during the period of May to July, which showed a slight increase in cases.

An analysis of the profile of people diagnosed with HVL revealed a higher percentage of occurrence among males (62%), with an age range of 0 to 12 years (53.1%), brown skin color (76.7%), education as “not applicable” (less than 4 years) (53%), urban area residence (89.2%), new cases (97.1%), and evolution towards recovery (86.5%), all at a level of statistical significance (*p*-value) (Table 1). Regarding the age range variable, it was observed that of the 369 children affected by the disease, 308 (44.96%) were aged between 0 and 4 years old.

As for clinical evidence, the most frequent symptoms were fever (96.7%), weakness (89.1%), paleness (85.8%), splenomegaly (76.3%), weight loss (74.4%), coughing (50.1%), and hepatomegaly (57.3%). 

**Figure 2:**
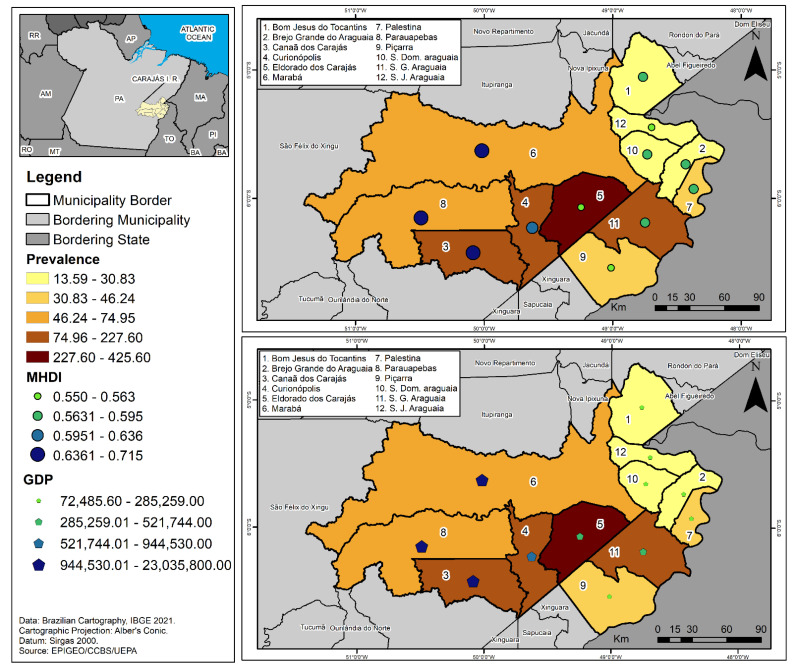
Human visceral leishmaniasis prevalence according to Gross Domestic Product and Human Development Index in the municipalities of the Carajás Integration Region, Pará, Brazil, for the period of 2011 to 2020.

An analysis of the HVL prevalence in the CIR according to GDP and MHDI showed that the municipalities of Eldorado dos Carajás, Canaã do Carajás, Curionopólis e São João do Araguaia, which had the region’s highest GDPs and different MDHI levels, presented very high and high prevalence of the disease, as shown in Figure 2.

**Figure 3:**
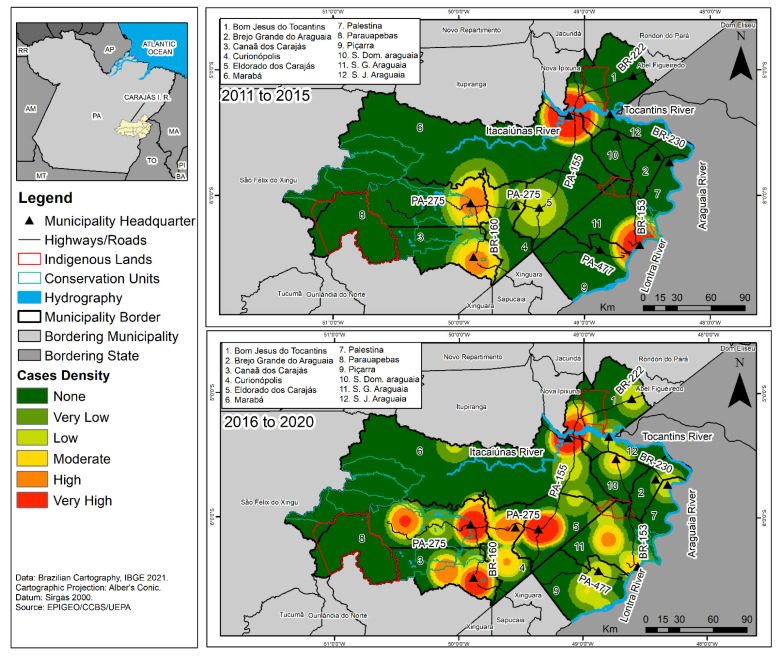
Cases density of human visceral leishmaniasis in the Carajás Integration Region, Pará, Brazil, for the period of 2011 to 2020.

With the KDE technique, it was possible to identify that the occurrence of HVL in the CIR presented a non-homogeneous pattern of case distribution. For the first period (2011 to 2015), a high density of leishmaniasis cases was observed in São Geraldo do Araguaia municipality, with evidence of vectorization along the PA-153 and BR-230. It was also noted that Marabá, Parauapebas, and Canaã do Carajás, respectively, presented medium and low case densities, while the other municipalities did not notify the disease in their territories, as shown in Figure 3.

During the second period (2016 to 2020), there was a significant increase in cases numbers in the CIR, with the disease spreading throughout the region. It was also possible to identify the presence of an epidemiological corridor marked by spatial dependence associated with the presence of uncontrolled settlements along the banks of the Tocantins, Itacaiuna and Vermelho rivers, and the highways that interconnect the municipalities in the CIR, especially along BR-230. A very high density of cases was observed in the mining municipalities of Marabá, Parauapebas, Canaã do Carajás, and Curianópolis, along with high and medium densities in most of the municipalities that make up the study area, as shown in Figure 3.

**Figure 4:**
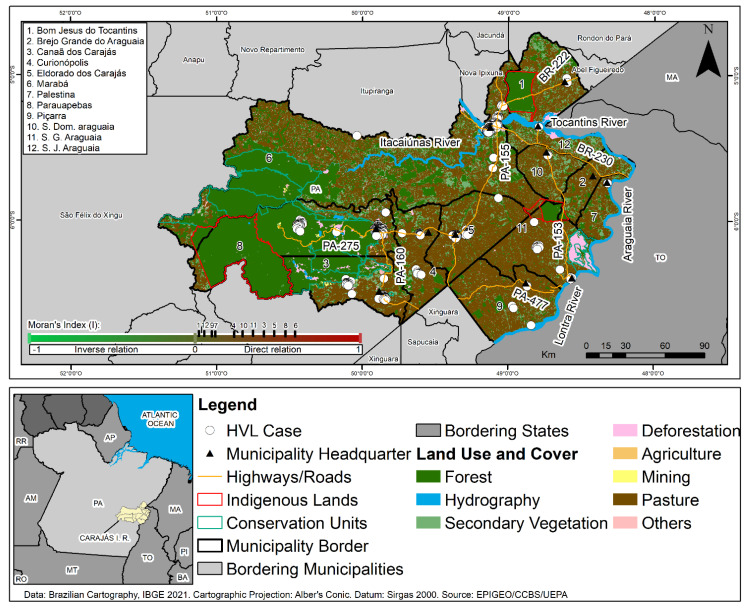
Spatial distribution of human visceral leishmaniasis cases, land use and cover, and interpretation of the Moran’s Index (I) spatial autocorrelation in the Carajás Integration Region, Pará, Brazil, for the period of 2011 to 2020.

The land use and cover map for the region showed very high percentages of pasture and small secondary forest fragments, as well as the presence of isolated forest remnants inside the CIR Conservation Units (UCs). Clusters of HVL cases were noted in Marabá, Parauapebas, Canaã do Carajás, and Curianópolis municipalities, where anthropic activities were identified related to economic activities, including mining, ranching, and urbanization, as seen in Figure 4.

Cases of HVL were notified inside the Carajás National Forest Conservation Unit, located in Parauapebas and Canaã dos Carajás municipalities, where mining activity was identified in bordering areas. Cases of the disease were also observed along the surroundings of the Igarapé Gelado and Araguaia Environmental Protection Areas and of edges the Mãe Maria Indigenous Lands of the Gavião Parkatejê People in the municipalities of Parauapebas, São Geraldo do Araguaia, and Bom Jesus do Tocantins, respectively, during the study period, as seen in Figure 4 (colocar a descrição de municipios na horizontal).

The spatial analysis of the relations between areas in the CIR municipalities that notified HVL cases and those that presented deforestation, using the Moran Global Bivariate index (I), showed significant spatial dependence relations between those variables. Thus, in Marabá (0.6201) and Parauapebas (0.5324), a strong and direct autocorrelation was noted, while in Eldorado dos Carajás (0.4417), Canaã dos Carajás (0.3541), São Geraldo do Araguaia (0.3017), Curionópolis (0.2025), São Domingos do Araguaia (0.2578), São João do Araguaia (0.0027), Piçarra (0.0029), Palestina do Pará (0.0033), and Bom Jesus do Tocantins (0.0021), there was a direct and weak autocorrelation (Figure 4).

**Figure 5:**
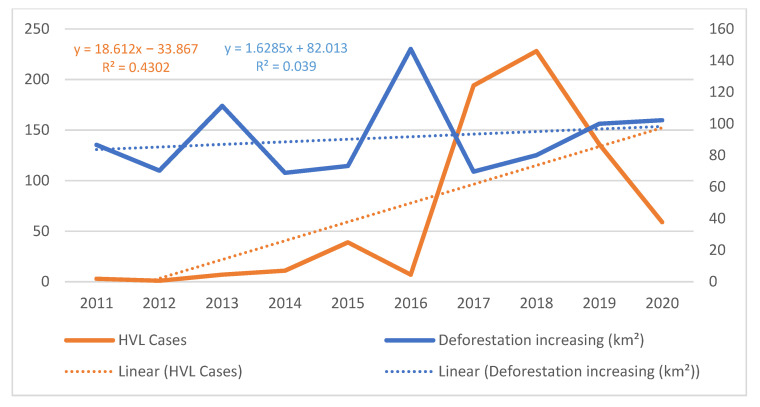
Relationship between the annual increase in deforestation and the number of HVL cases in the Carajás Integration Region, Pará, Brazil, for the period of 2011 to 2020.

The temporal analysis of HVL cases (R^2^ = 0.4302) and environmental degradation (R^2^ = 0.039) showed evidence of a direct relationship between these variables, with an increasing trend. It was thus possible to observe that during the study period, 2018 had the highest number of disease cases (228), with 2012 having the lowest (1). On the other hand, 2016 presented the highest deforestation increase (147.3 km²), while 2014 had the lowest (68.9 km²), as shown in Figure 5.

## 4. Discussion

HVL is a great public health problem in the state of Pará, especially in the CIR, due to its complex endemicity in the region’s municipalities. This suggests the presence of a multifactor dynamic associated with environmental, socioeconomic, demographic, and cultural characteristics of the region that favor the transmission chain and the establishment of this illness, such as inadequate housing conditions, human settlements in secondary forest areas, deforestation, intense migratory movement, lack of sanitation, and the presence of vectors and reservoirs [4,15,17]. 

In this context, the mining municipalities of Marabá and Parauapebas stand out as endemic areas with very intense transmission of HVL, being considered by the Pan-American Health Organization to be priority areas for measures to control the disease [3]. That fact indicates that the campaigns carried out in these territories and the health surveillance measures have not been in line with the goals determined for the PVCLV of the Ministry of Health [18].

Furthermore, to achieve its objectives in light of the different social, economic, cultural, and environmental contexts of the region, the PVCLV must consider the inevitable variations in specific circumstances of the same program. In other words, it must not assume that there is a homogeneous situation that may condition the occurrence of the disease. This is especially true when planning and implementing actions in areas that have development-focused projects that unsustainably exploit natural resources and are determining factors in social inequalities that leave the population vulnerable to risk factors for several diseases, including HVL [10,19]. 

The higher concentration of HVL during the months of May and July may be related to the characteristics of reproductive dynamics among phlebotomids. Thus, because of the environmental conditions during the rainy season (temperature, rainfall, and humidity) there is an increase in population density among those vectors, enabling greater probability of transmission of the disease [19,20,21,22]. Another important factor is the incubation period for the disease that occurs on average for 2 to 6 months, the time interval needed for the first symptoms to occur, as well as greater numbers and notifications of cases after the rainy season [1].

The epidemiological profile of men, children, and brown-skinned persons observed in this study is recurrent in other Brazilian territories, indicating the association of different behavioral, physiological, social, and populational factors related to those variables, including greater exposure to disease vectors, immature immunological systems (aggravated by nutritional deficiencies), and the ethnic origins of the Amazon population [23,24,25]. Generally speaking, there are no reports of an observable relation between ethnicity and susceptibility to infection, but instead a relation to the socioeconomic factors associated with these individuals [22].

The expressive number cases with education as “not applicable” may be related to the larger number of notifications of children who are not yet school-aged (less than 4 years-old) and living in family environments. This evidence indicates that transmission of the diseases occurs in households and their surrounding environments and points to the need for effective health surveillance measures, as called for in the PCLV [18,24]. Supporting this evidence, the presence of abandoned dogs was observed during the fieldwork, presenting a vulnerability to infection by the pathogen and other animals (chickens and pigs) being raised next to the households in the studied area.

Considering that HVL is a neglected disease [2,3], the significant number of cases found in this study involving children highlights the social inequities in child healthcare and the great risk this population faces in terms of sickness and death. Additionally, leishmaniasis infections in this age group often involve several psychosocial factors for children and their families, such as the need to seek out areas with greater health services, prolonged hospitalization, aggressive treatments, side effects of medicines, and changes to their daily routines.

The urban HVL transmission pattern suggests an association between four factors: rapid population growth, disorganized urbanization, spreading of urban areas without infrastructure, and socioenvironmental production of the disease based on the logic of social inequality and exclusion. Areas were observed that had unplanned human settlements, with inadequate housing, low socioeconomic power, and deposits of solid wastes in inappropriate locations. Those adverse conditions, along with accumulated organic wastes produced by domestic animals and the precarious basic sanitation conditions found in those pockets of poverty, may contribute towards maintaining breeding grounds and sources of food for the vectors and favor their adaptation to those environments [18,26,27]. 

The proportion of new cases observed indicates that an active HVL transmission process is occurring in the CIR municipalities and highlights the ineffectiveness of public health policies for monitoring and controlling the disease. This suggests the need for effective measures adapted to regional specificities that can contribute towards reducing HVL in the region, such as adequate treatment for cases in humans, canine surveillance with anti-canine visceral leishmaniasis vaccination, treatment for dogs with positive diagnoses and entomological surveillance, as well as health education [28] and socioeconomic development that seeks social equity in these territories.

The high percentage of cases ending in recovery (85.55%) suggests that the patients have received adequate care and treatment and that the strategies employed in Primary Health Care (PHC) for adherence to the treatment called for by the Brazilian Ministry of Health are presenting satisfactory results. However, treatment for HVL involves expensive medicines with possible side effects that may affect the quality of life of individuals affected by the disease. Therefore, prevention is still the best method to be used in controlling and possibly eradicating this disease [29,30]. 

Another factor that must be considered to better understand the HVL epidemiological scenario is the number of deaths that occurred during the study period. This finding illustrates the operational difficulties found by municipalities in the region in implementing public policies, such as actively search for new cases. Such policies can aid in early diagnosis of the disease and contribute towards breaking the chain of leishmaniasis transmission. These measures are very important for reducing lethality in territories where the disease is considered endemic, given that late diagnosis is one of the risk factors for death [28].

As for clinical evidence, most of the persons affected presented symptoms (fever, weakness, and weight loss) that are common in other parasitic diseases prevalent in the region (Chagas disease, malaria, schistosomiasis), which interferes in the clinical diagnosis of HVL [31,32]. However, the significant presence of signs, such as hepatosplenomegaly in endemic areas and the CIR municipalities, indicates late clinical diagnoses and the need for specific laboratory exams seeking a differential diagnosis of this pathology. Similar clinical manifestations were found in studies carried out in other regions of Brazil and the world [2,3,4,33]. 

The high and medium HVL density cases that occurred in the first period (2011–2015) along the PA-153 and BR-230 in São Geraldo do Araguaia, Marabá, Parauapebas, and Canaã dos Carajás suggests that those highways were an important means for the spatial vectorization of the disease, as they determined the sense and direction of the people flow, due to the macrodevelopmentalist model demands of the region, contributing towards the process of the dissemination through migratory processes that promote the circulation of humans and infected dogs. 

This fact is associated with the precarious social infrastructure and sanitation and economic conditions in the population clusters along those highways. These were implanted during the large agrarian colonization project in the Amazon in the 1970s, and are currently a major public health problem in the region. The disease vectorization process has also been observed in several studies, like Racan in the state of São Paulo and Miranda et al. in Pará [34,35]. 

Furthermore, the concentration of cases observed during the first period suggests that the social and administrative spatial reorganization of those territories, historically impacted by deforestation, natural resource extraction, and disordered urban expansion in remaining areas of forest, may have enabled contact between the population and phlebotomid species through invasion of their habitat [11,12,36,37,38]. Thus, the presence and circulation of vectors, reservoirs, and hosts in these areas may have favored the interaction between wild and domestic cycles of the disease and facilitated its establishment in semi-urban and urban areas [36,37,38]. 

The significant increase in HVL and its spread throughout most of the municipalities, observed during the second study period (2016–2020), including the formation of epidemiological corridors along the rivers and highways networks that interconnect the CIR, may be the result of structural changes of the demographic and territorial dynamic in the region, which is old, and new population clusters located along the Itacaiúnas and Tocantins rivers, PA-153 highway (that interconnects the North and Northeast regions of the country), and the BR-230 or Transamazon (built during the major agrarian colonization project in the Amazon) [7,8]. 

The high occurrence of HVL in municipalities with higher GDPs and HDIs suggests that the amount of wealth generated and the quality of life of the population in these territories, measured by these indicators, are not in line with the needs of sustainable local development, since this development model involves a character that is not restricted to socioeconomic factors and income concentration, but encompasses its environmental, cultural, equity, and public policy conditions regarding access to basic rights [39]. This paradox is illustrated by the epidemiological scenarios of the municipalities of Parauapebas and Marabá, which have, respectively, the second and third largest GDP in Pará and high and medium IDHMs. However, they are among the ten municipalities in the Americas with the highest occurrence of HVL cases [3,13].

Spatial analysis of types of land use and cover showed that CIR has a large percentage of its territory composed of deforested areas due to potentially degrading human impacts in the region, such as extensive beef cattle ranching (by companies and families), industrial mining, illegal “garimpagem”, and urbanization [7,10,11,12,13,17].

In this context, the high prevalence of LVH in different municipalities in the CIR, including territories related to the Serra Pelada mining (in Eldorado do Carajás and Curionópolis) and the largest open pit iron ore mine of South America (in Canaã dos Carajás), suggests that the socioeconomic relations existing in those areas polarize wealth, determining spatial poverty segregation and socioeconomic and territorial disparities. Thus, the complex nature of disease transmission goes beyond municipal borders and territorial dimensions and can be considered a paradox between the relation of economic growth and development in this region, located in the “Consolidated Settlement Arc” [40].

The occurrence of HVL cases inside of the Conservation Units (Flonas of Carajás) and in the surroundings of Indigenous Lands may be considered an indicator of socioenvironmental pressure resulting from productive activities and the land conflicts existing between the social actors in the region (placer miners, squatters, loggers, land-grabbers, miners, ranchers, farmers, and indigenous people) [11,12]. That fact highlights the ineffectiveness of the current set of public policies related to environmental degradation and the social inequalities in health.

Although cases of HVL have not been found in indigenous land, those areas have also had their natural environment degraded, with the production of risk factors for the disease. The lack of notification in those areas may be related to a potential epidemiological silence associated with several factors, such as ineffective differential diagnosis, difficulties in accessing health services, absence of inclusive indigenous health policies, and integrated health surveillance actions (control and health assistance, entomological and active search for infected reservoirs near the villages), with a view to empowering the traditional population regarding this issue [41].

However, despite this region being the largest iron ore producer with the second-largest cattle herd in the state of Pará [13], it may be stated that its socioeconomic dynamic is an antithesis of sustainable development, since there is a direct spatial correlation (weak and strong) between areas with significant numbers of HVL cases and the deforested areas. This fact demonstrates the cumulative and synergistic impacts resulting from the pressure of anthropic activities in the CIR municipalities. Furthermore, these anthropisms pressures the UCs’ mosaic that act as a physiological natural barrier, protecting the biodiversity and the different indigenous ethnic groups (Gavião Parkatejê People in the municipality of Bom Jesus do Tocantins; Suruís People, in the municipality of São Geraldo do Araguaia and Xikrin People in the municipality of Parauapebas).

During the process of economic growth in this region, primary forest areas have been deforested and converted into areas for agriculture, pastures, and mining, or turned into secondary forests. With that in mind, one may suggest that the deforestation caused by those activities and the stages of ecological succession for the vegetation also condition the production and establishment of the leishmaniasis transmission cycle in the region, as shown in Figure 4. Data from this study corroborate the findings by Scarro [19], who, in analyzing the impacts of deforestation on diseases in the Amazon, highlighted the need for inserting leishmaniosis incidences as a variable in the social costs for the environmental licensing of projects that involve suppressing vegetation, given that deforesting 1% of a given area leads to increases of 5% to 9% in the incidence of cases of the diseases [19].

## 5. Conclusions

This study analyzed the spatial distribution of HVL and its relations with epidemiological, socioeconomic, and environmental variables in the CIR, in the state of Pará. Although the epidemiological profile found also occurs in other areas of Brazil, a non-homogeneous distribution of the disease was found, with clusters of cases in municipalities that have environmental degradation associated with human activities that reproduce the perverse underlying logic of unsustainable development.

The occurrence of high socioeconomic indicators and high densities of HVL in some CIR municipalities showed that this region was socially and economically structured around unsustainable natural resources use that favored social and environmental production of the disease with disorganized land use and cover, including conservation units and indigenous lands. This situation influenced the epidemiological scenario observed for HVL, which has been affecting socially vulnerable populations in the region.

In this scenario, efficient and effective public policies are needed to improve the work, housing, health, education, and basic sanitation conditions, as well as development that considers social equity and respect for environmental limits. Furthermore, local measures for HVL monitoring and controlling, such as inclusive, permanent, and systematic health education, and distribution of repellents, mosquito screens, and nets, need to be prioritized by the State Health Secretariat.

## Figures and Tables

**Table 1:** Epidemiological profile of human visceral leishmaniasis in the Carajás Integration Region, Pará, Brazil, for the period of 2011 to 2020.

Variables	Category	Frequency	Proportion (%) n = 685	* *p*-Value
Sex	Female	249	36.35	<0.0001
	Male	436	63.65	
Age Range	Child	369	53.87	<0.0001
	Adolescent	58	8.47	
	Adult	232	33.87	
	Older Adult	26	3.79	
Ethnicity	White	63	9.20	<0.0001
	Black	52	7.59	
	Yellow	3	0.44	
	Indigenous	1	0.15	
	Brown-skin	517	75.47	
	Ignored	49	7.15	
Education	Illiterate	14	2.04	<0.0001
	Does not apply	304	44.38	
	Primary school	161	23.50	
	High school	58	8.47	
	Superior	3	0.44	
	Ignored	145	21.17	
Zone	Urban Area	604	88.18	<0.0001
	Periurban Area	2	0.29	
	Rural Area	71	10.36	
	Ignored	8	1.17	
Type of entry	New Case	597	87.15	<0.0001
	Recurrence	11	1.61	
	Transfer	7	1.02	
	Ignored	70	10.22	
Evolution	Abandonment	7	1.02	<0.0001
	Cure	586	85.55	
	Ignored	19	2.77	
	Death	63	9.20	
	Transfer	10	1.46	

n = Number of cases; * *p* < 0.05 (Chi-square, adherence).

## Data Availability

The data used in this study regarding the socioeconomic and environmental variables can be found at https://cidades.ibge.gov.br/ (accessed on 5 January 2022) and http://www.inpe.br/cra/projetos_pesquisas/dados_terraclass.php (accessed on 20 March 2022).
